# Enriched gestation activates the IGF pathway to evoke embryo-adult benefits to prevent Alzheimer’s disease

**DOI:** 10.1186/s40035-019-0149-9

**Published:** 2019-03-05

**Authors:** Enjie Liu, Qiuzhi Zhou, Ao-Ji Xie, Mengzhu Li, Shujuan Zhang, Hezhou Huang, Zhenyu Liuyang, Yali Wang, Bingjin Liu, Xiaoguang Li, Dongsheng Sun, Yuping Wei, Xiaochuan Wang, Qun Wang, Dan Ke, Xifei Yang, Ying Yang, Jian-Zhi Wang

**Affiliations:** 1 Department of Pathophysiology, School of Basic Medicine and the Collaborative Innovation Center for Brain Science, Key Laboratory of Ministry of Education of China for Neurological Disorders, Tongji Medical College, Huazhong University of Science and Technology, Wuhan, 430030 China; 2 Co-innovation Center of Neuroregeneration, Nantong University, Nantong, 226000 China; 3 Key Laboratory of Modern Toxicology of Shenzhen, Shenzhen Centre for Disease Control and Prevention, 8 Longyuan Road, Shenzhen, 518055 China; 4 Department of Pathology, the First Affiliated Hospital of Zhengzhou University, Zhengzhou, 450052 China

**Keywords:** Alzheimer’s offspring, Brain-derived neurotrophic factor, Gestational environment enrichment, Histone acetyltransferase, Insulin-like growth factor 1 receptor

## Abstract

**Background:**

Building brain reserves before dementia onset could represent a promising strategy to prevent Alzheimer’s disease (AD), while how to initiate early cognitive stimulation is unclear. Given that the immature brain is more sensitive to environmental stimuli and that brain dynamics decrease with ageing, we reasoned that it would be effective to initiate cognitive stimulation against AD as early as the fetal period.

**Methods:**

After conception, maternal AD transgenic mice (3 × Tg AD) were exposed to gestational environment enrichment (GEE) until the day of delivery. The cognitive capacity of the offspring was assessed by the Morris water maze and contextual fear-conditioning tests when the offspring were raised in a standard environment to 7 months of age. Western blotting, immunohistochemistry, real-time PCR, immunoprecipitation, chromatin immunoprecipitation (ChIP) assay, electrophysiology, Golgi staining, activity assays and sandwich ELISA were employed to gain insight into the mechanisms underlying the beneficial effects of GEE on embryos and 7–10-month-old adult offspring.

**Results:**

We found that GEE markedly preserved synaptic plasticity and memory capacity with amelioration of hallmark pathologies in 7–10-m-old AD offspring. The beneficial effects of GEE were accompanied by global histone hyperacetylation, including those at *bdnf* promoter-binding regions, with robust BDNF mRNA and protein expression in both embryo and progeny hippocampus. GEE increased insulin-like growth factor 1 (IGF1) and activated its receptor (IGF1R), which phosphorylates Ca^2+^/calmodulin-dependent kinase IV (CaMKIV) at tyrosine sites and triggers its nuclear translocation, subsequently upregulating histone acetyltransferase (HAT) and BDNF transcription. The upregulation of IGF1 mimicked the effects of GEE, while IGF1R or HAT inhibition during pregnancy abolished the GEE-induced CaMKIV-dependent histone hyperacetylation and BDNF upregulation.

**Conclusions:**

These findings suggest that activation of IGF1R/CaMKIV/HAT/BDNF signaling by gestational environment enrichment may serve as a promising strategy to delay AD progression.

**Electronic supplementary material:**

The online version of this article (10.1186/s40035-019-0149-9) contains supplementary material, which is available to authorized users.

## Background

Emerging evidence suggests that early life events during fetal development may trigger biochemical pathways that can cause AD in late life [1, 2]. High-fat diet-induced maternal obesity increases the risk of developing obesity and memory deficits in offspring [3, 4]. In contrast, maternal environmental enrichment during pregnancy accelerates the development of sensory and motor circuits in the fetus [5, 6] and improves the emotional and attentional reactivity of offspring to stresses in adulthood [7]. Maternal swimming or treadmill running or voluntary exercise during pregnancy enhances the memory capacity of offspring [8–11]. However, these scarce findings of positive effects on offspring are exclusively limited to healthy individuals and have not been extended to disease. Thus, we aimed to investigate whether GEE could alter the onset and**/**or disease severity in transgenic AD models, a devastating disorder that is currently not curable.

Studies indicate that environmental enrichment and voluntary exercise can increase adult neurogenesis [9, 12, 13] and promote neurotrophic and growth factors [14–16] in individuals per se exposed to the enrichment; however, the molecular mechanism underlying the parental influence on offspring is elusive. A recent study has shown that early life stress in the father improves the behavioral flexibility of offspring via epigenetic modification of histones [17]. Since the posttranslational modification of histones has a strong association with the burden of AD pathology [18, 19], we hypothesize that GEE may improve the offspring’s pathology and cognitive functions by modulating histone acetylation.

In the present study, we exposed pregnant 3 × Tg AD mothers to environment enrichment and measured the influences of GEE on the cognitive functions and AD-related pathologies in the offspring. We found that GEE preserved synaptic plasticity and spatial reference memory with attenuation of Aβ and tau pathologies in the adult offspring hippocampus. We also found that GEE increased insulin-like growth factor 1 (IGF1) and activated its receptor (IGF1R), of which the latter directly binds/phosphorylates Ca2+/calmodulin-dependent kinase IV (CaMKIV) to lead to the activation of histone acetyltransferase (HAT) with robust histone acetylation and BDNF production. Activation of the IGF1R/CaMKIV/HAT/BDNF pathway by GEE induced sustained chromosome remodeling, which underlies the beneficial effects of GEE.

## Methods

### Animals and antibodies

The 3 × Tg AD mice (Jackson Laboratory, Sacramento, CA, USA) were housed under a 12-h light/dark cycle with access to food and water at 25 °C. All animal studies were approved by the Ethics Committee of Tongji Medical College, Huazhong University of Science and Technology. The information for primary antibodies employed in the current study is listed in Additional file 1: Table S1.

### Cell culture, transfection, and drug treatment

The cells were cultured as reported previously [20]. In brief, HEK293 cells were cultured in DMEM supplemented with 10% (vol/vol) FBS (Thermo Fisher Scientific, Waltham, MA, USA) and grown at 37 °C in a humid atmosphere containing 5% (vol/vol) CO_2_. CaMKIV K75E plasmids were generous gifts from Tian-ming Gao (Southern Medical University, Guangzhou, China). After repairing the mutation site, CaMKIV K75E was used to generate EGFP-CaMKIV-WT (wild type CaMKIV) by PCR (Vazyme Biotech Co., Ltd.; China). The CaMKIV mutation (tyrosine − 136 and − 172 into phenylalanine (Y136F, Y172F)) was generated using the Mut Express II Fast mutagenesis kit following the manufacturer’s instructions (Vazyme Biotech Co., Ltd.; China). Transfection of EGFP-CaMKIV-WT, EGFP-CaMKIV-Y136F, EGFP-CaMKIV-Y172F or EGFP-CaMKIV-Y136F/Y172F in HEK293 cells was carried out using the Lipofectamine 2000 kit according to the manufacturer’s instructions. To explore the effect of IGF1, the cells were treated with IGF1 (100 ng/μl diluted with DMEM, 100–11, Peprotech, USA) for 2 h. PPP (1 μmol/l, AXL1717, HY-15494, MedChem Express, China), BMS-536924 (100 nmol/l, HY-10262, MedChem Express, China) or GDC-0994 (6 nmol/l, HY-15947, MedChem Express, China) was used to inhibit IGF1R or ERK. All inhibitor drugs used to treat the cell line were dissolved in DMSO (Sigma, USA), and the final concentration of the solvent was equal in all treatment groups.

### Environmental enrichment

Two male and four female 3 × Tg AD mice (2 m old) were mated for 2 days in each cage, and then the pregnant female mice were randomly distributed to either the home cage or GEE housing. Standard cages (33 cm × 18 cm × 14 cm) were used for the control mice, whereas large rat cages (55 cm × 34 cm × 20 cm) were used for the GEE. To increase the sense of novelty, the mice were arranged with constant access to toys of different shapes, sizes, materials and surface textures. Every 2 days, a set of 10–15 different toys was introduced into the cages to change the environment, and all the toys were moved prior to the female mice giving birth. The control group and GEE group consisted of 24 maternal female mice each, the GEE with C646 group contained 8 maternal female mice, and the GEE with BMS-536924 group had 8 maternal female mice. Female offspring were used in all fellow experiments.

### In vivo drug treatment

For inhibition of HAT in GEE, C646 (5 mg kg^− 1^ day^− 1^, S7152, Selleck, China) dissolved in 0.9% saline [17] was injected intraperitoneally into the pregnant mice starting at day 5 of the GEE exposure, and the injection was continued up to embryonic day 19 (E19). The mice in home cages or GEE controls were injected with saline. The offspring were reared in the home cage for 7 m, and then learning and memory ability were measured using the Morris water maze (MWM) or contextual fear conditioning. For inhibition of IGF1R in GEE, BMS-536924 (2 mg kg^− 1^ day^− 1^, dissolved in 30% PEG400 + 0.5% Tween80, HY-10262, MedChem Express, China) was injected intraperitoneally into the pregnant mice starting at day 5 of the GEE exposure, and the injection was continued up to E19; the controls were injected with the same volume of solvent.

### MWM test

The MWM test was performed as previously described [20]. In brief, the mice were trained to find a submerged platform hidden under water by following the constant cues outside the pool over 6 days. Before the training, we recorded the distance the mice swam in 60 s. Next, the mice underwent a daily session of three trials per day. During each trial, mice started facing the wall of the pool and found the hidden platform in 60 s, after which they were guided to and placed on the platform for 30 s. The swimming path and latency to locate the hidden platform were recorded in each trial by a digital device connected to a computer. On the eighth day, the mice were allowed to swim for 60 s freely with the hidden platform removed to test their memory. The percent time spent in the target quadrant and the numbers of platform quadrant crosses were recorded (Chengdu Taimeng Software Co. Lid, China).

### Contextual fear-conditioning test

The contextual fear-conditioning test was performed as previously described [21]. The mice were placed in the conditioning chamber for 3 min before an unconditioned stimulus (US) was administered, that is, a mild foot shock of 0.9 mA for 3 s, on the training day. Three sequential foot shocks at 3-min intervals were applied. All chambers were cleaned with 75% alcohol to eliminate any residual odor. To assess contextual memory, the mice were placed back in the training context 24 h post-training for 3 min without an electric foot shock. The activity and freezing behavior of the animals, which was defined as a complete absence of movement, were recorded by a video tracking system (Chengdu Taimeng Software Co. Lid, China).

### Western blotting

Western blotting was performed as reported previously [20]. Total proteins of hippocampi were extracted with RIPA lysis buffer (P0013B, Beyotime, China). Cytoplasm and nuclear proteins were extracted using the Nucl-Cyto-Mem Preparation Kit (P1201, Applygen Technologies Inc., China). The protein concentration was determined by the BCA method, and equal amounts of extracted proteins were separated by 10–12% SDS–polyacrylamide gel electrophoresis (SDS-PAGE) and transferred to nitrocellulose filter membranes. Then, the nitrocellulose membranes were blocked with 5% BSA for 1 h at room temperature. After blocking, the membranes were incubated with primary antibodies (Table S1). The bands were scanned and visualized using the Odyssey Infrared Imaging System (LI-COR biosciences, Lincoln, NE, USA).

### Immunohistochemistry

Immunohistochemistry was performed as reported previously [21]. The mice were anesthetized and immediately transcardially perfused with normal saline, followed by 4% paraformaldehyde (PFA). Brains were removed and post-fixed for another 48 h. Then, the brains were cryoprotected with 30% sucrose and frozen in OCT compound for cryostat sectioning. Brains were sliced coronally at a thickness of 30 μM using a freezing microtome (CM1900, Leica, Germany). The brain slices were soaked in PBS-3% H_2_O_2_–0.5% Triton for 30 min and then blocked with 3% BSA for another 30 min. The slices were incubated with anti-BDNF antibody (SC-546, Santa Cruz, Texas, USA) overnight at 4 °C, followed by a biotinylated secondary antibody for 1 h at 37 °C. Horseradish peroxidase-labeled antibodies were applied for 1 h at 37 °C to detect the immunoreaction, and staining was performed with DAB. The images were observed under a microscope (Nikon, 90i, Tokyo, Japan). For cell studies, the cells were cultured on coverslips and fixed with 4% paraformaldehyde for 30 min at room temperature, and then 1 μg/ml Hoechst 33258 (Sigma, St. Louis, MO, USA) was used for nuclear staining.

### Immunofluorescence

The brain slices were collected as described previously [21]. The slices were incubated overnight at 4 °C with primary antibody against acetyl-histone H3 (06–599, Millipore, CA, USA) or acetyl-histone H4 (06–866, Millipore, CA, USA), followed by Oregon green 488 as the secondary antibody for 1 h at room temperature. Hoechst 33258 (Sigma, St. Louis, MO, USA) was used for nuclear staining. The images were observed with a laser confocal microscope (710, Zeiss, Germany).

### Immunoprecipitation

E19 hippocampi or cultured cells were homogenized on ice in lysis buffer at 4 °C for 30 min and centrifuged at 12,000 g for 10 min. A total of 300 μl of supernatants containing approximately 600 μg of total proteins was incubated overnight with rotation at 4 °C with 10 μg of anti-CBP, anti-CaMKIV, anti-GFP, or anti-IGF1R antibody, followed by the addition of protein A + G agarose at 4 °C for 2 h. The agarose beads were washed three times and re-suspended in 60 μl of SDS loading buffer. The precipitates were analyzed by western blotting using anti-phosphoserine, anti-CBP, CaMKIV, pCaMKIV, or anti-phosphorylated tyrosine. To explore the interaction of IGF1R and CaMKIV, 600 μg total proteins were incubated overnight with rotation at 4 °C with 10 μg of anti-CaMKIV antibody (Santa Cruz, 136,249, USA) or anti-IGF1R antibody (Proteintech, 20,254, China), and the precipitates were analyzed by western blotting using anti-CaMKIV or IGF1R.

### Real-time PCR

Total RNA was extracted using TRIzol reagent (Invitrogen, Carlsbad, CA, USA), and the reverse transcription reagent kit (RR037, Takara) was used to obtain cDNA. The published primers complementary to each *bdnf* noncoding exon I–V were used to assay the level of expression of each individual transcript of *bdnf* [22]. RT–PCR was performed using a StepOnePlus Real-Time PCR Detection System (Life Technologies, NY, USA).

### Chromatin immunoprecipitation (ChIP) assay

The ChIP analysis was performed according to published methods and Upstate Biotechnology ChIP kit (17–371, Millipore, USA) protocols using the following antibodies: anti-acetyl-histone H3 (06–599, Millipore, USA); anti-acetyl-Histone H4 (06–866, Millipore, USA) and mouse immunoglobulin-G (12–371B, Millipore, USA). DNA fragments in immunoprecipitated samples were quantified by quantitative real-time PCR with published primers designed around the putative promoter regions of *bdnf* PI–V [22].

### Electrophysiology

Mice were deeply anesthetized with 40 mg kg^− 1^ pentobarbital, and the brains were immediately removed and immersed in ice-cold oxygenated artificial cerebrospinal fluid (ACSF; 2.0 mM KCl, 125 mM NaCl, 1.2 mM MgSO_4_, 26 mM NaHCO_3_, 1.2 mM KH_2_PO_4_, 2.5 mM CaCl_2_ and 11 mM glucose). Parasagittal sections (300 μm) were cut using a vibrating microtome (Leica VT1000S, Leica Biosystems) at 4–5 °C in ACSF, and the sections were pre-incubated in oxygenated ACSF at 30 °C for at least 1 h. Then, one section was placed in a chamber with an 8 × 8 microelectrode array (Alpha MED Sciences, Panasonic) and kept submerged in artificial cerebrospinal fluid (aCSF; 1–2 ml min^− 1^), The ACSF temperature in the recording chamber was maintained at 34 °C by a heat exchanger. The MED64 system (Alpha MED Sciences, Panasonic, Japan) was used to record the fEPSPs in CA1 neurons by stimulating the Schaeffer fibers from CA3. LTP was induced by applying three trains of high-frequency stimulation (HFS; 100 Hz, 1-s duration) separated by 20 s.

### HAT and HDAC activity assays

The activity of HAT was assayed using a HAT activity assay kit (p-4003, Epigentek, NY, USA), and the activity of HDACs was assayed using a HDAC activity assay kit (P-4034, Epigentek, USA), following the manufacturer’s instructions.

### Sandwich ELISA for Aβ

The hippocampi of GEE and control offspring were rinsed twice in PBS and homogenized in RIPA buffer (P0013D, Beyotime Biotechnology, China) containing a protease inhibitor cocktail (P8340, Sigma, USA). RIPA samples were sonicated briefly and centrifuged at 12,000 g for 10 min. The levels of Aβ1–42 and Aβ1–40 in the supernatant (1.5 μg μl^− 1^) were measured using a sandwich ELISA kit (E-EL-H0543, Elabscience, China) following the manufacturer’s instructions.

### Golgi staining

The mice were anesthetized as mentioned above and perfused intracardially with 300 ml of 0.9% saline containing 0.5% sodium nitrite, followed by 300 ml of 4% formaldehyde solution and the Golgi dye solution (5% potassium dichromate, 4% formaldehyde, and 5% chloral hydrate) for 1 h. After being perfused, the brains were dissected into 4 mm × 4 mm sections and transferred to a vial containing Golgi dye solution for 5 days in the dark, followed by a solution containing 1% silver nitrate once a day for 3 days. Serial 50-μm-thick sections of the brain were obtained using a vibrating microtome (Leica, VT1000 S, Germany).

### Statistics

Data are expressed as mean ± s.e.m. and were analyzed using commercial software (GraphPad Prism, GraphPad Software, Inc., La Jolla, CA; SPSS version 21.0 for Windows, SPSS Inc., Chicago, IL, USA). Two–way ANOVA, one–way ANOVA or Student’s t–test was used to determine different means among groups. The level of significance was set at *p <* 0.05.

## Results

### GEE preserves offspring cognitive capacity and synaptic plasticity in AD mice

**Fig. 1:**
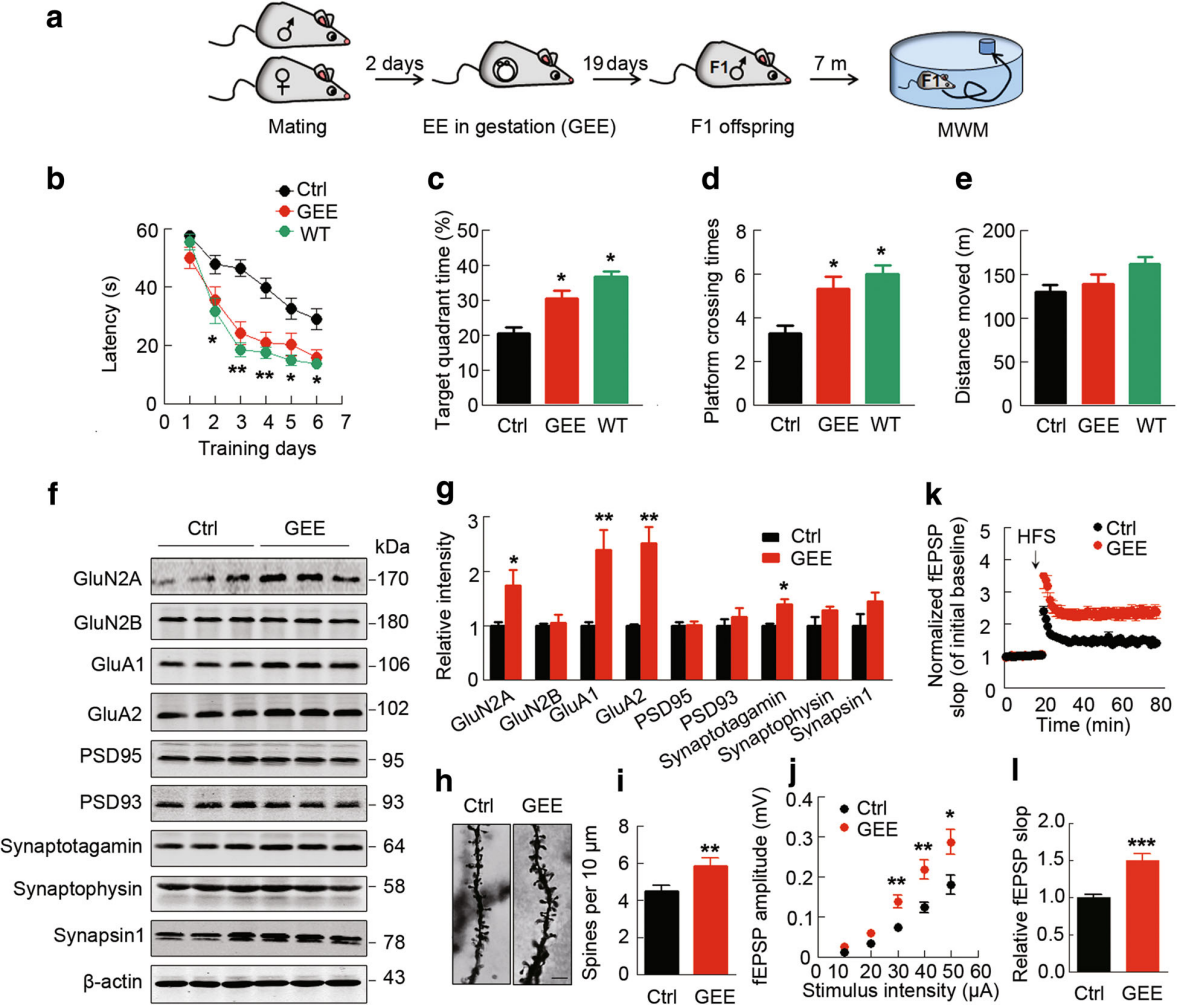
GEE preserves cognitive capacity and synaptic plasticity in offspring of 3XTg AD mice. (**a**) The experimental procedure: female 3 × Tg AD mice were mated with males, and then the pregnant females were housed in an enriched (GEE) or standard environment (Ctrl). After about 19 days of pregnancy, the EE was removed. The GEE or Ctrl F1 AD offering and age-matched wildtype (WT) mice were raised in a standard environment from weaning until 7 m of age, and then spatial learning and memory were tested in the Morris water maze (MWM). (**b-e**) GEE preserves spatial learning (**b**) and memory (**c** and **d**) without changing motor function (**e**) in AD offspring (WT, *n* = 8, Ctrl, *n* = 12, GEE, n = 12, from at least 4 independent litters, two–way ANOVA, Bonferroni’s post hoc test). (**f,g**) GEE increases protein levels of GluN2A, GluA1, GluA2 and synaptotagmin without changing GluN2B, PSD93, PSD95, synaptophysin and synapsin-1 in the hippocampus of 7 m-old offspring (from at least 3 independent litters, unpaired t-test with Welch’s correction). (**h**) Representative images of dendritic spines in CA1 neurons measured by Golgi staining (scale bars, 10 μm). (**i**) GEE increases spine density in the CA1 subset (at least 14 neurons from 6~7 mice were analyzed in each group, unpaired t-test with Welch’s correction). (**j-l**) GEE enhances basal synaptic transmission, as shown by an increased input–output response with potentiation of LTP indicated by an increased slope of the evoked fEPSP; the increase was still significant at 60 min after high frequency stimulation (HFS) (*n* = 10–12 hippocampal slices from 6~7 mice in each group, unpaired t-test with Welch’s correction). Data are presented as the mean ± s.e.m. *P < 0.05, **P < 0.01 vs. Ctrl

To explore whether GEE could affect cognitive functions in AD offspring, we first created a GEE model (described in the Methods section). Then, the offspring were reared in the home cage for 7 m, and learning and memory ability were measured by the Morris water maze (MWM) and contextual fear-conditioning (FC), respectively (Fig. 1a; Additional file 2: Figure S1).

During the 6-day learning test in the MWM, the GEE offspring could locate the hidden platform in the water maze much faster than the controls, and the difference was already very significant at the second day (Fig. 1b). During the memory test, the platform was removed at day 8, and the GEE offspring showed a significantly increased target duration (*p <* 0.05) and target crossings (*p <* 0.05) (Fig. 1c and d), suggesting a potentiation of spatial memory. GEE did not change the offspring’s motor function compared with the home cage controls (Fig. 1e). These data suggest that GEE can improve offspring’s spatial learning and memory in AD transgenic mice.

In the contextual fear-conditioning test in separate sets of mice, we did not observe a significant difference between the two groups during training, but significantly increased freezing (*p <* 0.01) was identified in the GEE offspring during the memory test carried out the next day after training (Additional file 2: Figure S1), suggesting an improved contexture memory in the offspring by GEE.

To explore the mechanisms underlying the improved cognitive functions, we measured the memory-related synaptic plasticity in the offspring. By western blotting, we observed that GEE significantly increased the levels of GluN2A (*p <* 0.05), GluA1 (*p <* 0.01), GluA2 (*p <* 0.01) (postsynaptic proteins) and synaptotagmin (*p <* 0.05) (presynaptic protein), without changing GluN2B, PSD93, PSD95, synaptophysin and synapsin-1 (Fig. 1f and g). Simultaneously, the spine density and number of spines were significantly increased in GEE offspring compared with the controls (*p <* 0.01) (Fig. 1h and i). By ex vivo brain slice electrophysiological recordings, we found that GEE facilitated basal synaptic transmission, as shown by an increased input-output (I-O) curve with enhanced LTP in the hippocampus (Fig. 1j, k and l). These data suggest that the facilitated synaptic plasticity may underlie the improved memory by GEE.

### GEE upregulates BDNF/TrkB and histone acetylation at bdnf promoters

**Fig. 2:**
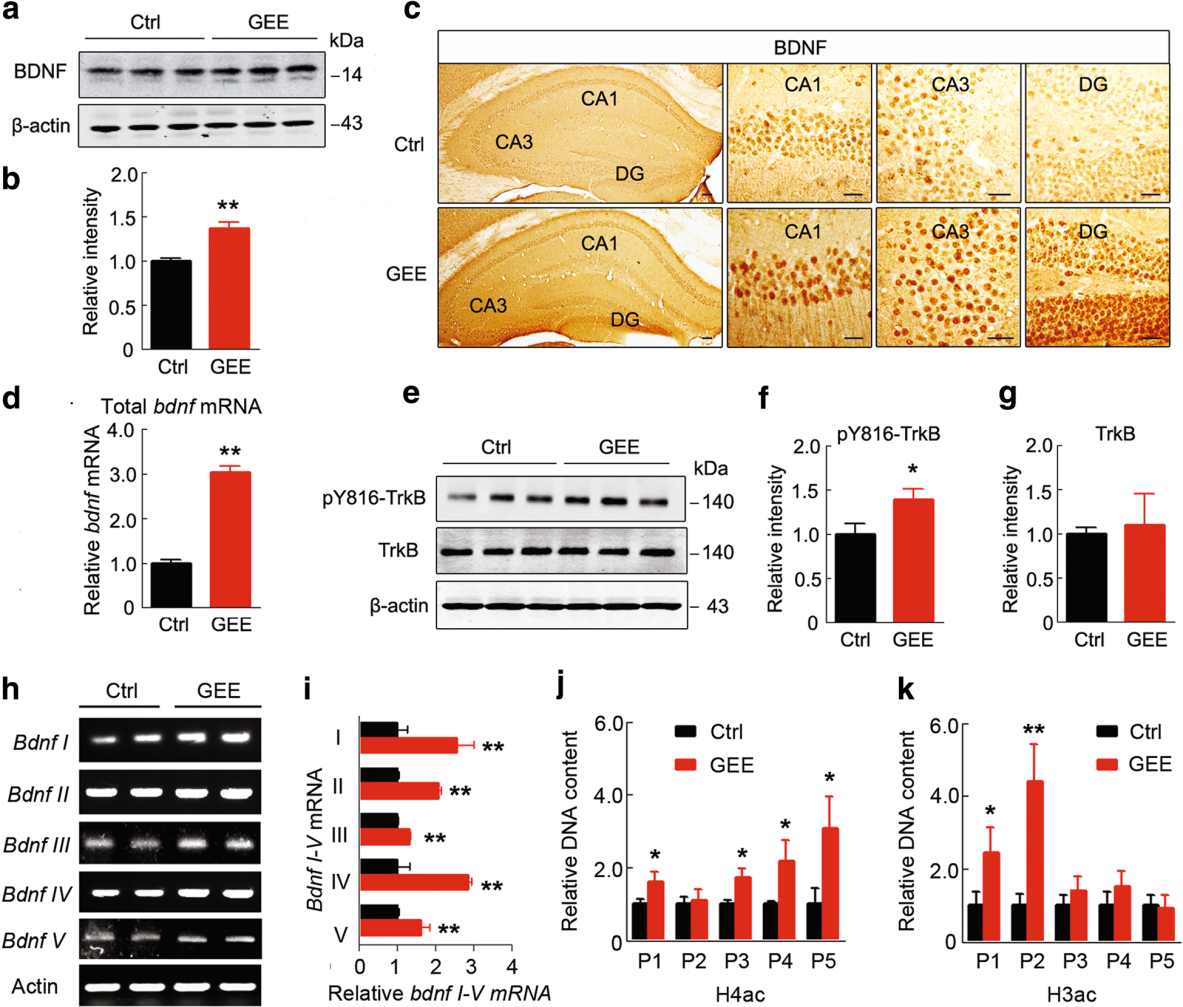
GEE activates BDNF/TrkB signaling with increases in acetylated histone binding to *bdnf* gene promoters in AD offspring. (**a-d**) GEE increases BDNF protein and mRNA levels in 7-m-old offspring hippocampus, as measured by western blotting, immunohistochemical staining (scale bars, 50 μm) and qRT-PCR. (**e-g**) GEE increases TrkB phosphorylation at Tyr816 without changing the total protein level. (**h,i**) GEE increases *bdnf* mRNA transcript variants in 7-m-old offspring hippocampus, as measured by agarose gel electrophoresis. (**j,k**) GEE increases acetylated histone 4 (H4ac) and H3ac at the indicated *bdnf* promoter regions, as measured by the CHIP assay. Data are presented as the mean ± s.e.m. of at least 3 independent litters of mice, unpaired t-test with Welch’s correction, *P < 0.05, **P < 0.01 vs. Ctrl

BDNF is crucial for neuronal activity and synaptic functions, and a previous study has suggested that mice exposed to EE show increased BDNF expression [23]. Therefore, we measured whether BDNF upregulation was also involved in GEE offspring. We found that both protein and mRNA levels of BDNF were significantly increased in the GEE offspring (*p <* 0.01) (Fig. 2a-d) with increased phosphorylation of TrkB at tyrosine-816 (*p <* 0.05) (Fig. 2e-g), indicating that activation of BDNF signaling may underlie the increased expression of synaptic proteins by GEE.

To explore the mechanisms underlying the augmented BDNF expression, we first analyzed the mRNA level of *bdnf* variants in 7-m-old offspring hippocampus. We found that five *bdnf* transcripts (I-V) were significantly increased in the GEE offspring (*p <* 0.01) (Fig. 2h and i). To further explore the mechanism by which the corresponding promoters might be regulated by GEE to lead to the increased *bdnf* expression, we measured histone acetylation at 5 different *bdnf* promoter-binding regions using chromatin immunoprecipitation (ChIP). The results showed that GEE increased histone (H4) acetylation at *bdnf* P1, P3, P4 and P5, and H3 acetylation at *bdnf* P1 and P2 (Fig. 2j and k). These data suggest that GEE may stimulate BDNF expression by enhancing histone acetylation at its specific promoter-binding regions.

### GEE promotes histone acetylation and BDNF expression by activating HAT in offspring

**Fig. 3:**
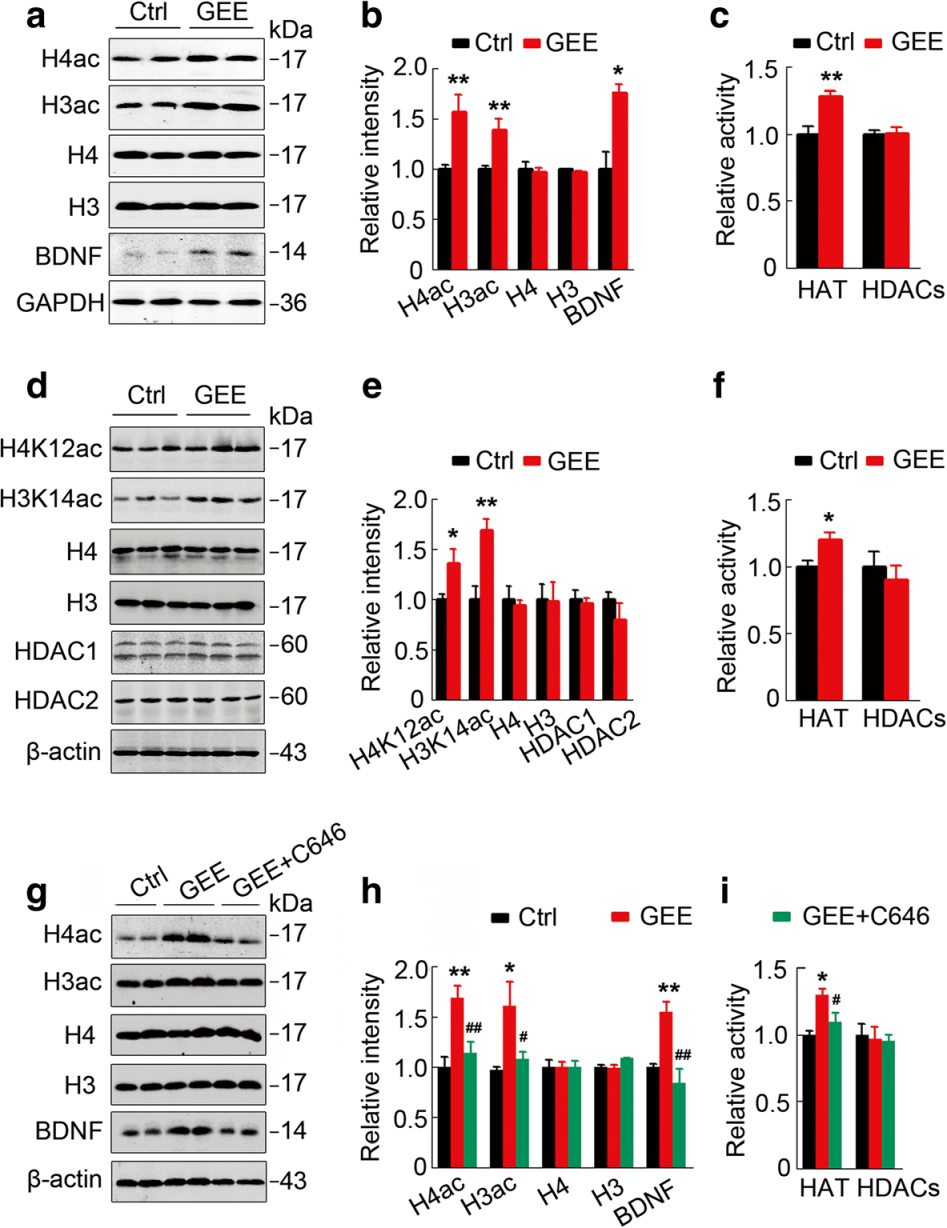
HAT activation mediates GEE-potentiated histone acetylation and BDNF expression in AD offspring. (**a,b**) GEE increases acetylation of histone 4 (H4ac) and histone 3 (H3ac) without changing the total levels of H4 and H3 in the embryo (E19), as measured by western blotting (*n* = 3, unpaired t-test). (**d,e**) GEE increases H4 acetylation at K12 (H4K12ac) and H3 at K14 (H3K14ac) in 7-m-old offspring hippocampus without changing the total levels of H4 and H3 (n = 3, unpaired t-test). (**c,f**) GEE activates histone acetyltransferase (HAT) without changing histone deacetylases (HDACs) in embryos (Ctrl, *n* = 6, GEE, n = 8, unpaired t-test) and 7-m-old offspring hippocampus (*n* = 9 per group, unpaired t-test). (**g-i**) Simultaneous inhibition of HAT by intraperitoneal injection of C646 during pregnancy abolishes GEE-induced H4 and H3 acetylation and as well as BDNF upregulation (*n* = 4~6, one–way ANOVA). Data are presented as the mean ± s.e.m. of at least 3 independent litters of mice. *P < 0.05, **P < 0.01 vs. Ctrl; ^#^*P* < 0.05, ^##^*P* < 0.01 vs. GEE

To investigate upstream factors of the enhanced acetylation at *bdnf* promoter regions, we measured the activities of HAT and HDACs in the offspring embryos and the adult hippocampus. First, we confirmed that GEE significantly increased levels of total acetylated H3 (*p <* 0.01) and H4 (*p <* 0.01) (H3ac and H4ac) and K14- and K12-acetylation of H3 (*p <* 0.01) and H4 (*p <* 0.05) (H3K14ac, H4K12ac) in E19 embryonic brains (Fig. 3a and b) and 7-m-offspring hippocampus (Fig. 3d and e), respectively. The increases in H3ac and H4ac were also detected by immunofluorescence staining in 7-m-offspring hippocampus with GEE exposure (Additional file 3: Figure S2). Simultaneously, BDNF expression was upregulated, while the total protein levels of H3 and H4 were not changed (Fig. 3a, b, d and e). By activity assay and western blotting analyses, we found that GEE significantly upregulated HAT activity without changing the expression and activity of HDACs (Fig. 3c-f). These data together indicate that GEE may potentiate the expression of neurotrophic proteins (such as BDNF) via activating HAT-mediated histone acetylation during embryonic development, thus improving memory in the adult offspring.

To validate the critical role of the embryonic activation of HAT in GEE-induced histone acetylation and activation of BDNF/TrkB signaling, we intraperitoneally injected HAT inhibitor (C646 5 mg kg^− 1^) into the pregnant mice starting at day 5 after exposure to EE. We confirmed that the activity of HAT and the levels of histone acetylation and BDNF expression were all increased with the upregulation of BDNF in E19 embryonic brains after GEE exposure, while simultaneous inhibition of HAT to roughly normal levels abolished the GEE-induced histone acetylation and BDNF upregulation (Fig. 3g-i). Injection of C646 did not change the weight of the embryos (Additional file 4: Figure S3). These data confirm the role of HAT activation in GEE-induced histone acetylation and BDNF expression in the offspring.

Furthermore, by correlation analysis, we observed a positive association between GEE-improved spatial memory and HAT activity (Additional file 5: Figure S4a, b and c). In contrast, no correlation was detected between hippocampal HDACs and spatial performance after GEE treatment (Additional file 5: Figure S4d, e and f). These data further support the critical role of HAT activation in the GEE-induced memory improvement.

### GEE activates HAT by upregulating CaMKIV and ERK in offspring

**Fig. 4:**
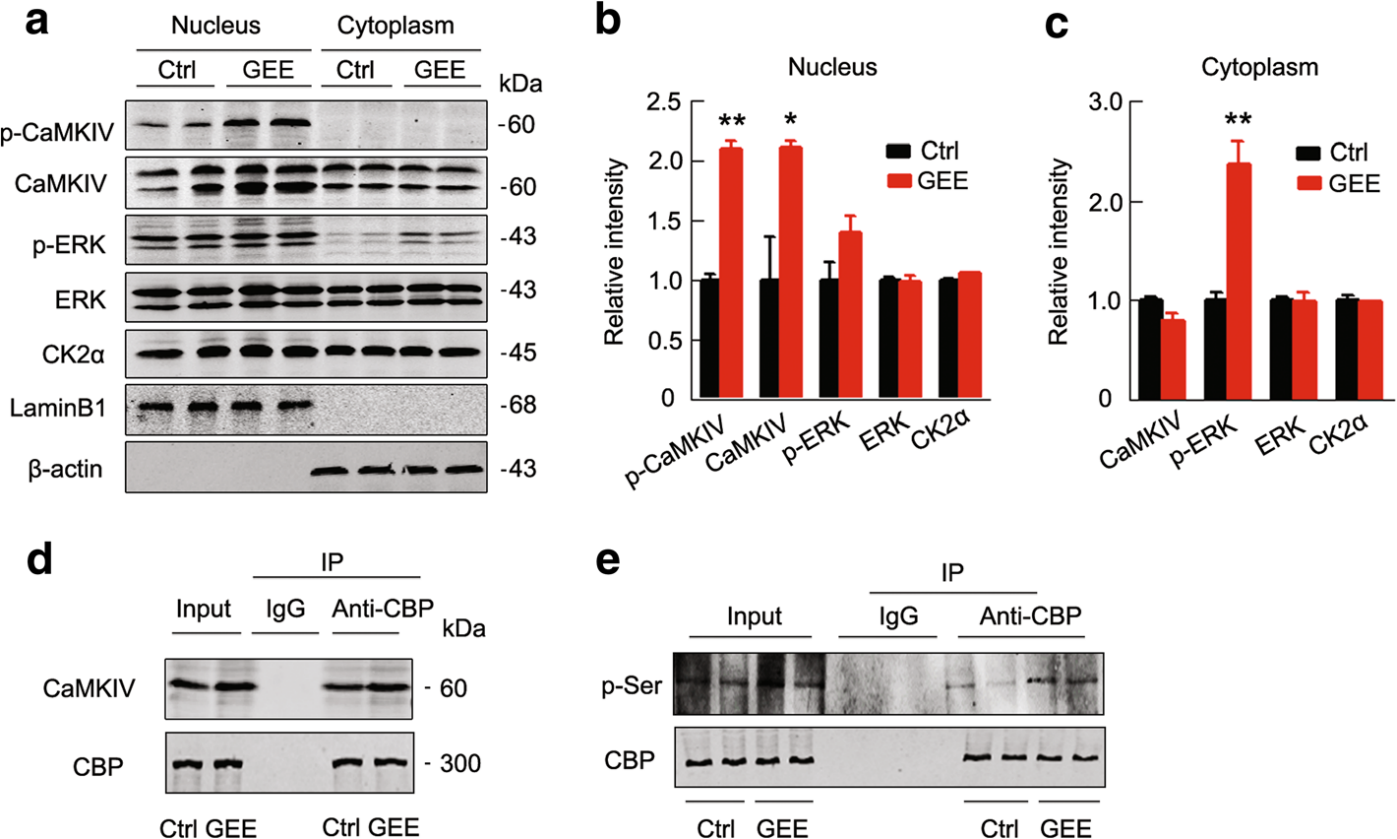
GEE upregulates CaMKIV and ERK and increases CaMKIV-CBP binding in the E19 AD embryo. (**a-c**) GEE increases total and the phosphorylated CaMKIV in the nuclear fraction of E19 embryo brain, as measured by western blotting. (**d**) GEE increases the association of CaMKIV with CREB-binding protein (CBP), as measured by co-immunoprecipitation. (**e**) GEE increases CBP phosphorylation, as measured by immunoprecipitation using anti-CBP and western blotting using anti-pSer antibody. Data are presented as the mean ± s.e.m. of at least 3 independent litters of mice, unpaired t-test, *P < 0.05, **P < 0.01 vs. Ctrl

Previous studies have suggested that CaMKIV and ERK can activate HAT [24, 25]. Therefore, we measured the expression and activity-associated phosphorylation of the kinases in cytoplasmic and nuclear fractions. We found that both total (*p <* 0.05) and phosphorylated levels of CaMKIV (*p <* 0.01) were significantly increased in the nuclear fractions of the E19 offspring brain after GEE exposure in which p-CaMKIV was exclusively detected in the nuclear fraction (Fig. 4a and b). p-ERK was also significantly increased (*p <* 0.01) in the cytoplasmic fraction, while total ERK was not changed in either the nuclear or cytoplasmic faction after GEE (Fig. 4a-c). In addition, we measured the expression of CK2α, the upstream kinase of HDACs, but no change was found (Fig. 4a-c). By co-immunoprecipitation, we found that GEE increased the association of CaMKIV with CREB-binding protein (CBP), an important subtype of HAT (Fig. 4d) with enhanced CBP phosphorylation (Fig. 4e). These data indicate that the upregulation of CaMKIV and ERK can be upstream of the GEE-induced HAT activation.

### Upregulation of IGF1/IGF1R controls GEE-potentiated CaMKIV/ERK-HAT-BDNF signaling

**Fig. 5:**
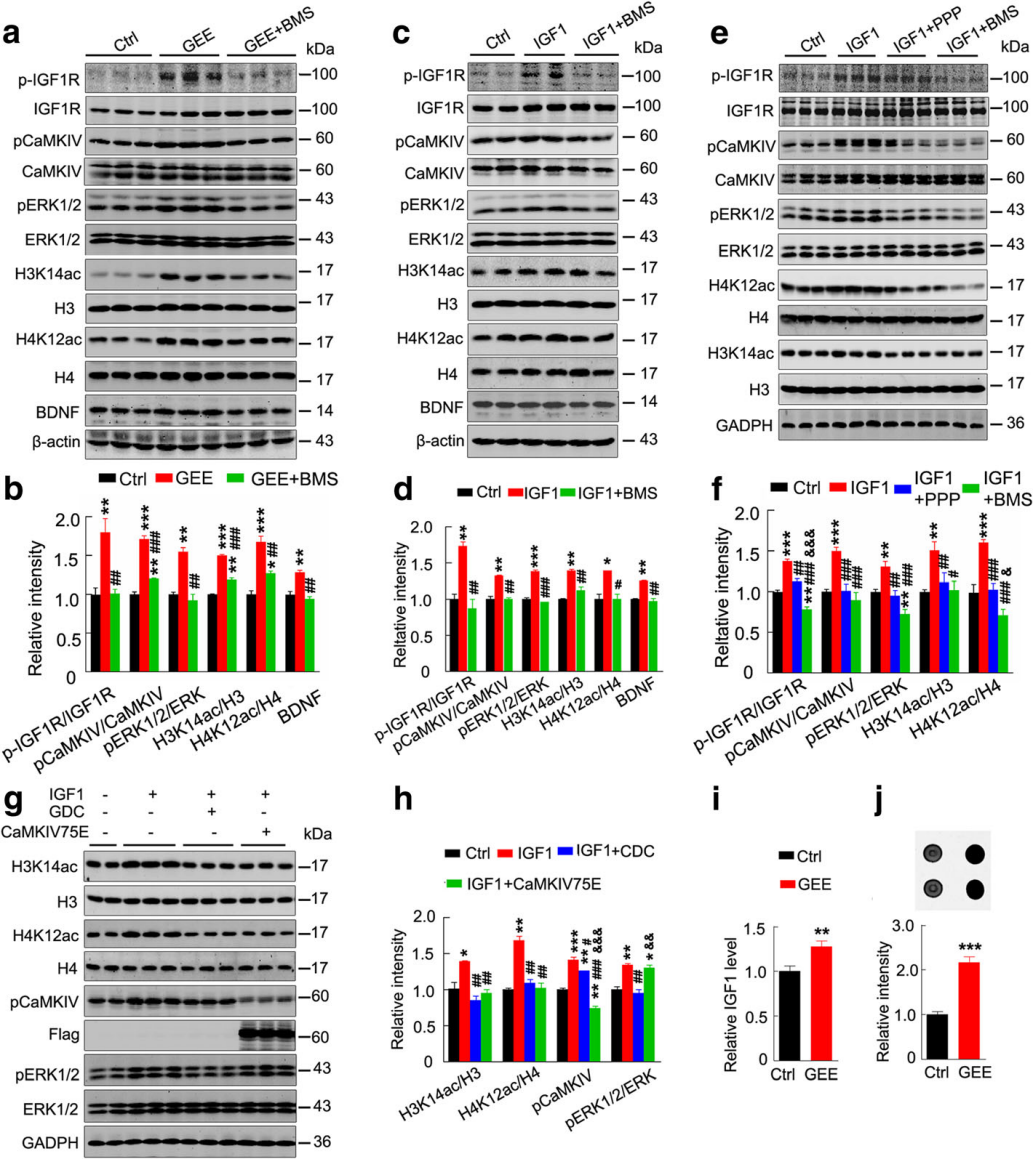
GEE activates IGF1R-CaMKIV/ERK signaling and thus promotes histone acetylation and BDNF expression. (**a,b**) In 3XTg AD mice, GEE activates IGF1R with upregulation of CaMKIV/ERK/HAT/BDNF signaling in their offspring (7 m old), while simultaneous inhibition of IGF1R by BMS abolishes GEE-induced histone acetylation, CaMKIV and ERK activation, and BDNF expression (n = 3, one–way ANOVA, *P < 0.05, **P < 0.01, ***P < 0.001 vs. Ctrl; ^#^*P* < 0.05, ^##^*P* < 0.01, ^###^*P* < 0.001 vs. GEE). (**c,d**) In 3-m-old wildtype mice, brain lateral ventricle infusion of IGF1 activates IGF1R with an upregulation of CaMKIV/ERK/HAT/BDNF signaling, while simultaneous inhibition of IGF1R by BMS attenuates these effects (n = 3, one–way ANOVA, *P < 0.05, **P < 0.01,***P < 0.001 vs. Ctrl; ^#^*P* < 0.05, ^##^*P* < 0.01, ^###^*P* < 0.001 vs. IGF1). (**e, f**) In HEK293 cells, upregulation of IGF1R by IGF1 treatment activates CaMKIV and ERK with increased histone acetylation, while inhibition of IGF1R by PPP or BMS attenuates these effects (n = 6, one–way ANOVA, *P < 0.05, **P < 0.01, ***P < 0.001 vs. Ctrl; ^#^*P* < 0.05, ^##^*P* < 0.01, ^###^*P* < 0.001 vs. IGF1; & *P* < 0.05, &&*P* < 0.01, &&&*P* < 0.001 vs. IGF1 + PPP). (**g, h**) In HEK293 cells, simultaneous inhibition of ERK or CaMKIV by CDC0994 or by expressing the inactive CaMKIVK75E abolishes IGF1-induced histone acetylation (n = 3, one–way ANOVA, *P < 0.05, **P < 0.01, ***P < 0.001 vs. Ctrl; ^#^*P* < 0.05, ^##^*P* < 0.01, ^###^*P* < 0.001 vs. IGF1; & *P* < 0.05, &&*P* < 0.01, &&&*P* < 0.001 vs. IGF1 + CDC0994). (**i**) GEE increases the plasma level of IGF1 in the pregnant host, as measured by ELISA (n = 8, unpaired t-test, **P < 0.01 vs. Ctrl). (**j**) GEE increases the IGF1R level in E19 embryo brain, as measured by dot blotting (*n* = 5, unpaired t-test, ***P < 0.001 vs. Ctrl). Data are presented as the mean ± s.e.m. and offspring tissue collected from at least 3 independent litters of mice

A previous study has shown that retinal IGF1R is activated in normal mice subjected to EE [26]. Here we found that GEE significantly activated IGF1R with an upregulation of CaMKIV/ERK/HAT/BDNF signaling in the offspring of AD mice, while simultaneous inhibition of IGF1R by intraperitoneal injection of BMS-536924 (BMS) abolished the GEE-induced upregulation of CaMKIV and ERK activity, histone acetylation and BDNF expression (Fig. 5a,b). Furthermore, brain lateral ventricular infusion of IGF1 mimicked the GEE effects, i.e., activation of IGF1R with upregulation of CaMKIV/ERK and histone acetylation; while simultaneous inhibition of IGF1R by BMS attenuated the IGF1 effects in naïve mice (3 m old) (Fig. 5c,d). In HEK293 cells, upregulation of IGF1R by addition of IGF1 to the culture medium (100 ng/μl) activated CaMKIV and ERK with increased histone acetylation, while inhibition of IGF1R by picropodophyllin (PPP, 1 μmol/L) or BMS-536924 (BMS, 100 nmol/L) attenuated CaMKIV and ERK activation (Fig. 5e,f). Furthermore, simultaneous inhibition of ERK or CaMKIV by GDC0994 (GDC, 6 nmol/L) or expression of the CaMKIV-K75E [20] (a dominant negative mutant of CaMKIV) to block the function of endogenous CaMKIV abolished IGF1-induced histone acetylation and CaMKIV/ERK activation (Fig. 5g,h). GEE also increased host (mother) plasma IGF1 levels with an increase in IGF1 in E19 embryo brain (Fig. 5i,j). Taken together, these data suggest that the upregulation of IGF1/IGF1R controls CaMKIV/ERK-HAT-BDNF signaling during GEE.

### CaMKIV phosphorylation at Tyr136/172 is essential for IGF1R-promoted histone acetylation

**Fig. 6:**
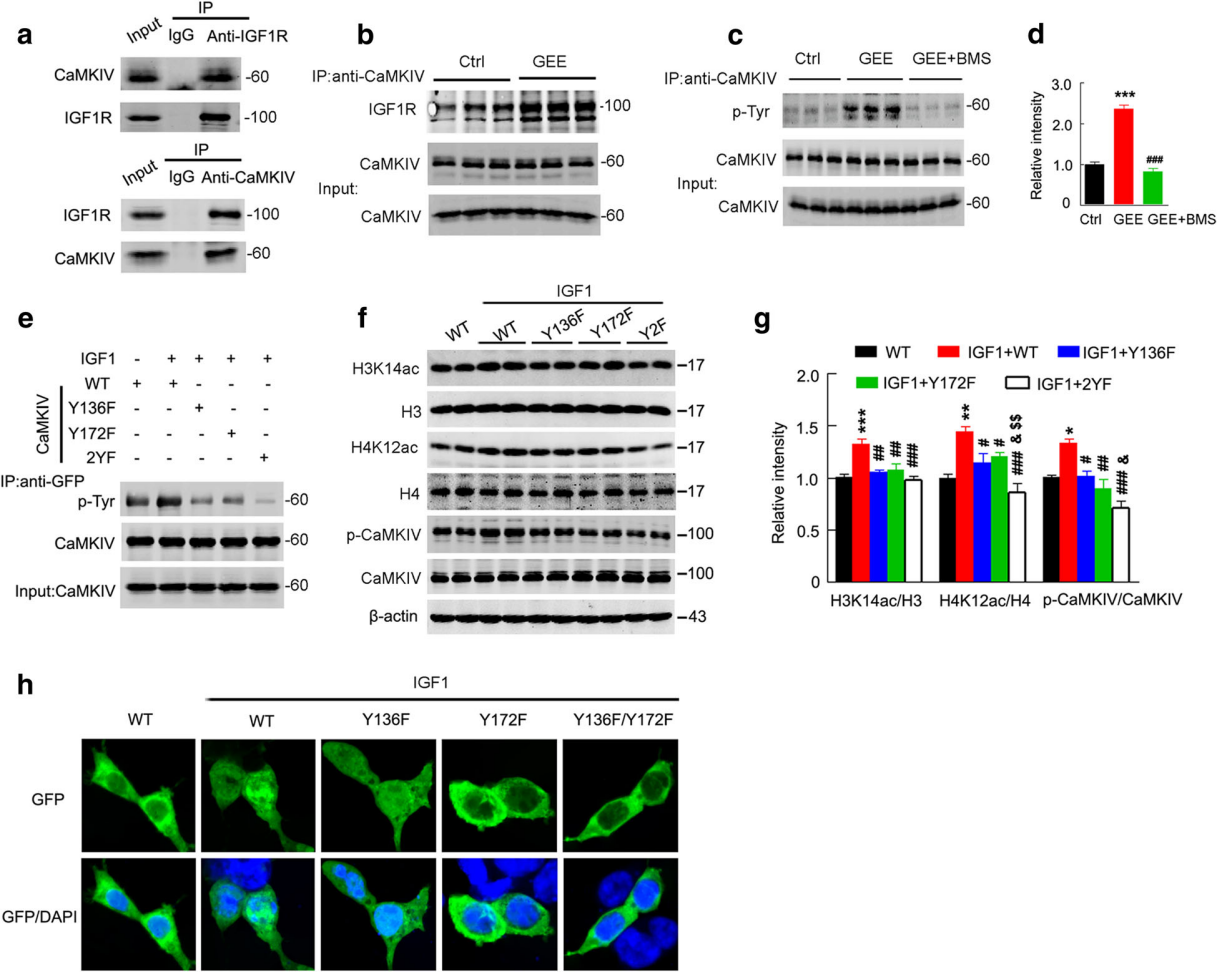
CaMKIV activation mediates IGF1-induced histone acetylation. (**a, b**) IGF1R interacts with CaMKIV, and GEE enhances the association of IGF1R with CaMKIV in E19 embryo extracts, as measured by co-immunoprecipitation and western blotting. (**c, d**) Inhibition of IGF1R reverses GEE-increased CaMKIV phosphorylation at tyrosine residues (n = 3, one–way ANOVA, ***P < 0.001 vs. Ctrl; ^###^*P* < 0.001 vs. GEE). (**e**) Mutation of CaMKIV at Y136 and Y172 dose-dependently decreases the IGF1-induced CaMKIV phosphorylation at tyrosine residues. HEK293 cells transfected with EGFP-CaMKIV-WT, EGFP-CaMKIV-Y136F, EGFP-CaMKIV-Y172F or EGFP-CaMKIV-Y136F/Y172F for 24 h were subsequently treated with IGF1 (100 ng/μl) for 2 h, and the cell extracts were immunoprecipitated using anti-GFP and blotted with anti-p-Tyr and CaMKIV antibodies. (**f, g**) Mutation of CaMKIV at Y136 and Y172 abolishes IGF1-induced histone acetylation and CaMKIV phosphorylation. HEK293 cells transfected with EGFP-CaMKIV-WT, EGFP-CaMKIV-Y136F, EGFP-CaMKIV-Y172F or EGFP-CaMKIV-Y136F/Y172F for 24 h were subsequently treated with IGF1 (100 ng/μl) for 2 h, and levels of histone acetylation and CaMKIV phosphorylation in cell extracts were measured by western blotting (n = 4, one–way ANOVA, *P < 0.05, **P < 0.01,***P < 0.001 vs. WT; ^#^*P* < 0.05, ^##^*P* < 0.01, ^###^*P* < 0.001 vs. IGF1 + WT; & *P* < 0.05, &&*P* < 0.01, &&&*P* < 0.001 vs. IGF1 + Y136F; $$ *P* < 0.01 vs. IGF1 + Y172F). (**h**) IGF1 induces nuclear translocation of CaMKIV, while mutation of CaMKIV at Tyr172 abolishes the effect of IGF1. Data are presented as the mean ± s.e.m.

IGF1R may induce CaMKIV phosphorylation through AMPAR-dependent L-type calcium channels in adult neurons [27]. In HEK293 cells that barely express endogenous AMPAR, we also detected the regulation of IGF1R on CaMKIV, suggesting the involvement of other mechanisms. To test this hypothesis, we performed a co-immunoprecipitation. We observed an association of IGF1R with CaMKIV, and GEE enhanced the association in E19 embryos (Fig. 6a, b). Furthermore, we detected hyperphosphorylated CaMKIV at tyrosine residues after GEE, which could be reversed by IGF1R inhibition (Fig. 6c, d). To predict the phosphorylation site(s) of CaMKIV, we used the NetPh3.1 Server software and found that Y136 and Y172 could be phosphorylated by IGF1R. For this analysis, we expressed site-specifically mutated CaMKIV plasmids (EGFP-CaMKIV-Y136F or EGFP-CaMKIV-Y172F or EGFP-CaMKIV-Y136F/Y172F) in HEK293 cells for 24 h and then treated the cells with IGF1 (100 ng/μl) for 2 h. We observed that mutation of CaMKIV at Y136/Y172 dose-dependently blocked IGF1-induced CaMKIV phosphorylation with reduced histone acetylation (Fig. 6e-g). Immunofluorescence data showed that IGF1 induced nuclear translocation of CaMKIV, while mutation of at Tyr172 abolished IGF1-induced nuclear translocation of CaMKIV (Fig. 6h). These data suggest that IGF1R may phosphorylate/activate CaMKIV to stimulate histone acetylation.

### GEE reduces Aβ and tau pathologies in adult offspring hippocampus

**Fig. 7:**
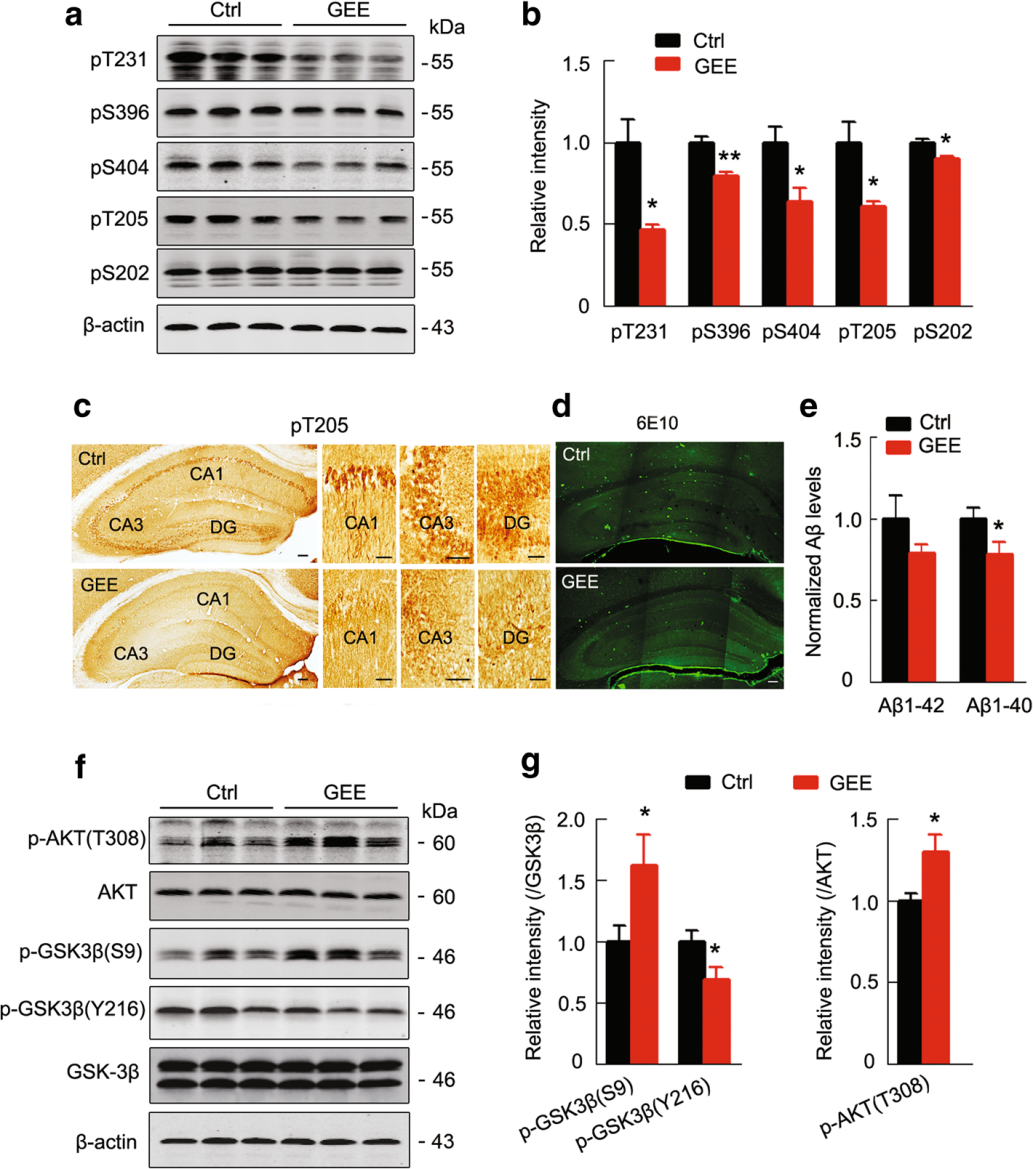
GEE reduces tau and Aβ pathologies in 10-m-old AD offspring hippocampus. (**a-c**) GEE attenuates tau hyperphosphorylation at multiple AD-associated sites, as measured by western blotting and immunohistochemical staining (scale bar, 50 μm). (**d,e**) GEE decreases the Aβ1–40 level with reduced Aβ plaques in the cortex and hippocampus, as measured by ELISA and immunofluorescent staining (scale bars, 50 μm, from at least 4 independent experiments, unpaired t-test). (**f,g**) GEE activates AKT, as shown by increased phosphorylation at Thr308 (pT308AKT) with inhibition of GSK-3β determined by increased pS9GSK-3β and reduced pY216GSK-3β. The phosphorylation level of AKT and GSK-3β was normalized against total AKT and GSK-3β, respectively. Data are presented as the mean ± s.e.m. of at least 3 independent litters of mice, unpaired t-test (**e** and **g**), *P < 0.05, **P < 0.01 vs. Ctrl

The 3 × Tg AD mice showed significant Aβ and tau pathologies. We observed that GEE exposure significantly attenuated tau hyperphosphorylation at multiple AD-associated sites in the hippocampus, as measured by western blotting and immunohistochemical staining (Fig. 7a-c, Additional file 6: Figure S5). GEE exposure also decreased the A β1–40 level (*p <* 0.05), with significantly reduced Aβ plaques in 10-m-old offspring cortex and hippocampus (Fig. 7d and e). GEE activated AKT with inhibition of the downstream GSK-3β (*p <* 0.05) (Fig. 7f and g), which not only partially explained the mechanisms underlying the attenuated tau phosphorylation but also indicated an activation of cell survival signaling [28]. These data together suggest that GEE may improve brain metabolism to arrest the dominant gene mutation-induced pathologies in the offspring, which can contribute to the improved synaptic plasticity and memory capacity.

## Discussion

Based on current protocols in molecular biology, autosomal dominant hereditary AD genes (such as APP, PS1/2) are feasibly sieved out in the parental generations for diagnosis. However, to date, no effort has been made to examine this type of early diagnosis of hereditary AD. The major hindrance is due to the lack of efficient interventions, i.e., why should those very young people (carrying dominant AD mutant genes) be told that they or their children will suffer from early onset AD if nothing good can be done to help them?

**Fig. 8:**
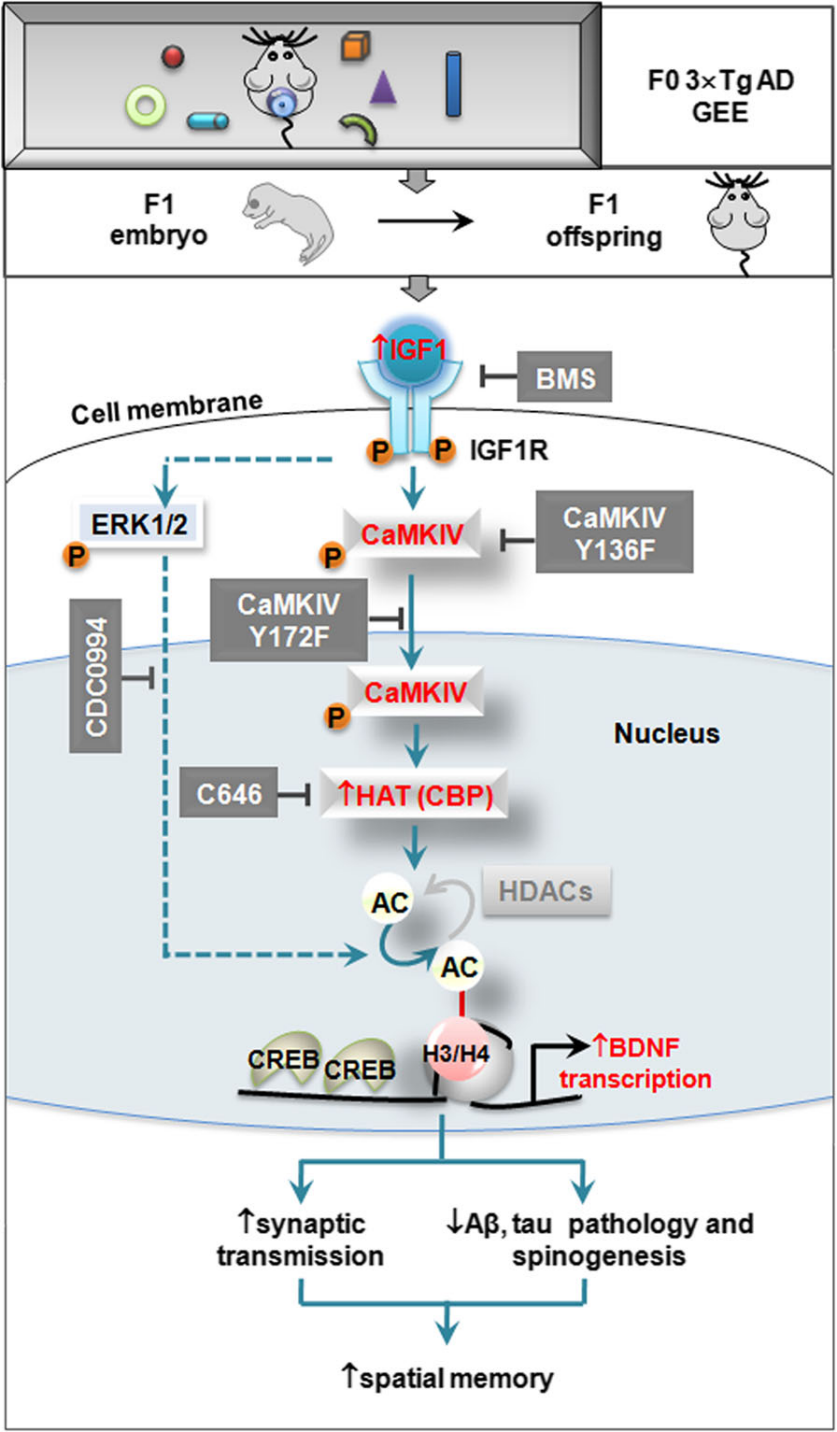
Proposed working model. Gestational environment enrichment (GEE) in mothers (F0) upregulates IGF1/IGF1R in offspring (F1, embryo and the adults), which in turn phosphorylates/activates ERK and CaMKIV. The phosphorylated pY172-CaMKIV is translocated to the nuclear fraction where it phosphorylates HAT (CBP), thus inducing hyperacetylation at *bdnf* promoters and upregulating BDNF expression. BDNF preserves synaptic plasticity and improves AD pathologies, which eventually leads to an improved spatial memory capacity. Blockade of HAT, CaMKIV, ERK, and IGF1R abolishes the beneficial effects of GEE

Building the brain reserve before the onset of dementia could be a promising strategy to prevent AD. The enriched environment (EE) is a well-known type of cognitive stimulation that has been tested in many adult AD models. The discrepant readouts raise the possibility that decreasing brain dynamics along with ageing might limit the efficiency of EE. Thus, we shifted our focus to the embryonic period, one of the most dynamic periods of life, and employed GEE in AD mice. We found that GEE significantly preserved synaptic plasticity and memory capacity with remarkably ameliorated pathologies in 7–10-m-old offspring, and the molecular mechanism involved an upregulation of IGF1/IGF1R/CaMKIV/HAT/BDNF signaling (Fig. 8). These findings reveal that maternal GEE can arrest the offspring’s pathological and behavioral progressions in the AD model. As there is currently no cure for AD, our findings provide a feasible and promising non-drug-involved approach for intervention in hereditary AD at a very early stage.

Previous studies have focused on the influence of EE on individuals per se who have received EE (F0), and paradoxical findings have been obtained. Some studies have shown that EE has significant benefits in animal models of neurodegenerative diseases, including AD [29–32]; long-term or short-term exposure to EE can improve synaptic plasticity [33, 34] and thus mitigate cognitive deficits [32, 33]; EE can reduce amyloid deposition and tau hyperphosphorylation [32]. In contrast, data have also been obtained to support EE-induced plaque formation in a double transgenic mouse model of AD [35], or a failure of EE to rescue working memory deficits or neuron loss in APP/PS1KI mice [36]. The reason for this discrepancy is currently not fully understood. We speculate that different ages may contribute to the distinct effects of EE, in addition to the genetic variability of the AD animal models employed in these studies. In the present study, we observed a significant beneficial influence of GEE on AD offspring compared with age-matched controls.

The mechanism underlying the improved cognition by GEE is currently not clear. In normal rats, offspring from maternal swimming or treadmill running show an enhanced short-term memory with significantly increased *bdnf* mRNA in the hippocampus [8, 10, 37]. Maternal voluntary exercise during pregnancy promotes BDNF expression in male offspring with enhanced learning, while simultaneous inhibition of BDNF action (TrkB inhibitor) abolishes the effects of maternal exercises on memory retrieval [11]. Maternal enrichment accelerates fetal retinal development with an increase in IGF, which can be inherited by the offspring from the mother [6]. These data strongly suggest that the upregulation of neurotrophic factors and altered neural development are involved in exercise-induced cognitive improvement. The BDNF level has been found to be decreased in the brain of AD postmortem samples and AD animal models [38–41], and defective BDNF is involved in the synaptic dysfunction and cognitive impairment in AD [39, 41–43], while the upregulation of the BDNF pathway can reverse these phenomena [44–48]. In the present study, we also observed an upregulation of BDNF signaling in AD offspring after GEE. Although decreased BDNF levels are well recognized in the brain of AD patients and in animal models [38–41], at present, no medicine targeting BDNF or its receptor, TrkB, has been clinically shown to exert precise anti-AD effects, which may be due to difficulties related to entry of the drugs into the brain and their short half-life. Thus, the search for a continuous and effective strategy to elevate brain BDNF or activate its signal is of great significance for the treatment of AD. BDNF expression is regulated epigenetically, for example, by DNA methylation and histone acetylation [49]. The offspring from both maternal and parental EE showed a global decrease in DNA methylation in the hippocampus and frontal cortex with an increase in exploratory behavior in the open field test [50]. Histone acetylation is a key mechanism in regulating *bdnf* transcription [51, 52], and increases in histone acetylation enhance BDNF mRNA expression [53, 54]. Here we found that GEE increased histone acetylation at multiple *bdnf* promoter regions, which was accompanied by global histone hyperacetylation both in the embryo and the offspring hippocampus, and the inhibition of HAT abolished GEE-enhanced BDNF expression. GEE specifically activates CaMKIV, a crucial kinase for HAT activation, without altering HDACs or the upstream CK2α. These data suggest that GEE can induce the embryo-adult elevation of BDNF via histone hyperacetylation and upregulation of *bdnf* transcription by activating CaMKIV-HAT signaling.

One of the important novel findings of the present study is that GEE-upregulated IGF1, as a key contributor, drove the activation of CaMKIV-HAT signaling. We systemically demonstrated that GEE activated the CaMKIV/ERK/HAT/BDNF signaling pathway by upregulating IGF1/IGF1R. GEE enhanced the association of IGF1R with CaMKIV and, thus, increased CaMKIV phosphorylation and its nuclear translocation by IGF1R. The activation of retinal IGF1R in normal mice who received EE exposure has been reported [26], but our current data demonstrated that GEE could upregulate the IGF1/IGF1R signaling pathway in AD offspring. We also revealed a site-specific phosphorylation of CaMKIV by IGF1R, thus providing a novel mechanism by which IGF1R can induce CaMKIV phosphorylation through AMPAR-dependent L-type calcium channels [27].

BDNF activates a variety of signaling cascades, including PI3K/Akt [55], and activation of BDNF/TrkB promotes neuronal survival and synaptic plasticity largely through Akt [56–58]. Increasing evidence suggests that the PI3K/Akt pathway is inhibited in the AD brain [59] or by Aβ exposure [60, 61]. GSK-3β is downstream of Akt [62] , which is responsible for AD-like tau hyperphosphorylation [63]. We found that GEE activated BDNF/TrkB signaling in AD offspring, which can inhibit GSK-3β by activating Akt and thus attenuate tau hyperphosphorylation. Previous studies have shown that BDNF can reduce the Aβ level in AD transgenic mice [64, 65], and here we found that GEE could decrease the level of Aβ in AD offspring.

Aerobic exercise training increases the hippocampus/brain volume and improves cognitive function in aging humans [66–68]. Randomized clinical trials with longitudinal follow-up studies have also shown that physical activity can improve cognitive function in older adults at risk for AD or prevent the onset of dementia [69, 70]. The human studies have also indicated that maternal physical and/or cognitive exercise is not only beneficial for the amniotic fluid, placental viability and body fat deposition, but it also affects neurodevelopment in the offspring [71–73]. High levels of leisure-time physical activity (e.g., jogging, aerobics, yoga, weight-lifting) during pregnancy are associated with increased vocabulary in offspring [74]. Exercise increases the plasma levels of IGF1 and BDNF with maternal hormonal changes in pregnancy [75]. The level of IGFBP-2, an inhibitory factor of IGF, is increased [76, 77], while insulin and IGF1 responsiveness are reduced in the AD patients [78]. Studies also show that a decrease in the plasma IGF1 level can predict cognitive decline in AD [79], and the elevation of IGF1 mediates the environment enrichment- and exercise-induced beneficial effects on the central nervous system [80–83]. The enriched environments used in the current study included the running wheel, toys and gregarious living, which mimic increased exercise, an enriched living environment, and an enhanced social interaction. These environmental enrichments, together with the measurement of plasma IGF1, may be applied for human studies in the future.

In the progression of AD neurodegeneration, compensation involves the previously acquired functional reserve, while building this reserve relies on cognitive stimulation before the onset of disease [84]. The protocol used in the present study consisted of the idea that individuals who continually receive intellectually stimulating activities maintain higher and prolonged intellectual abilities [85, 86], and those with good physical fitness show improved memory in old age. More interestingly, compared with the uncertain benefits of EE in adults, GEE in our study could produce embryo-adult benefits via the IGF1R/CaMKIV/HAT/BDNF pathway to prevent AD, which may be a promising method for AD prevention.

## Conclusions

In summary, this is the first report to show that GEE efficiently delays AD progression via inducing a sustained stimulation of the IGF1R/CaMKIV/HAT/BDNF pathway, which not only provides a promising strategy for early intervention of AD but also reveals novel molecular mechanisms for the lasting enhanced brain plasticity induced by GEE.

## Additional files

Additional file 1: **Table S1.** Antibodies employed in this study. (DOCX 206 kb)

Additional file 2: **Figure S1.** GEE improves spatial memory in offspring, as measured by contextual fear-conditioning. GEE was conducted as shown in Fig. 1a. (**a**) F1 offspring were raised in a standard environment from weaning until 7 m of age when memory was tested using contextual fear conditioning (FC). (**b**) GEE improved memory without changing learning ability in the offspring. Ctrl, *n* = 16, GEE, *n* = 11, unpaired t test with Welch’s correction. **P<0.01 versus Ctrl. Data are presented as the mean ± s.e.m. (DOCX 113 kb)

Additional file 3: **Figure S2.** GEE increases H3 and H4 acetylation in the offspring hippocampus. Representative immunofluorescence images stained using acetylated H3 (H3ac) and H4 (H4ac) in 7-m-old offspring hippocampal subsets (CA1, CA3 and DG). Scale bars, 50 μm. (DOCX 522 kb)

Additional file 4: **Figure S3.** Inhibition of HAT during GEE exposure has no effect on embryo weight. Embryos were weighed at E19 after GEE exposure with or without C646 treatment (HAT inhibitor). No differences in embryo weights were detected among the groups. *n* = 8–15 per group, one–way ANOVA, Tukey’s multiple comparisons test. Data are presented as the mean ± s.e.m. (DOCX 32 kb)

Additional file 5: **Figure S4.** Hippocampal HAT but not HDAC activity is positively correlated with spatial memory performance after GEE treatment. (**a-c**) Pearson analyses show the positive correlation of HAT activity with time spent in the target quadrant and platform crossings in the MWM test, and the freezing time in FC (for original data, see Fig. 1 and Figure S1). (**d-e**) No correlation was detected between hippocampal HDAC activity and partial memory performance after GEE treatment. Data are presented as the mean ± s.e.m. (DOCX 183 kb)

Additional file 6: **Figure S5.** GEE decreases tau phosphorylation in offspring hippocampus. Representative immunohistochemical images of phosphorylated tau at Ser396 and Thr231 in hippocampal subsets (CA1, CA3 and DG). Scale bars, 50 μm. (DOCX 2457 kb)

## Abbreviations

AD: Alzheimer’s disease
Aβ: Amyloid β
BDNF: Brain derived neurotrophic factor
CaMKIV: Ca^2+^/calmodulin-dependent kinase IV
CBP: CREB-binding protein
ChIP: Chromatin immunoprecipitation
FC: Contextual fear-conditioning
GEE: Gestational environment enrichment
HAT: Histone acetyltransferase
IGF1: Insulin-like growth factor 1
IGF1R: Insulin-like growth factor 1 receptor
I-O curve: Input-output curve
MWM: Morris water maze
SDS-PAGE: SDS–polyacrylamide gel electrophoresis
US: Unconditioned stimulus
