# Supplementary material for: Enriched gestation activates the IGF pathway to evoke embryo-adult benefits to prevent Alzheimer’s disease
Source: Transl Neurodegener. 2019 Mar 5;8:8. doi: 10.1186/s40035-019-0149-9 (PMC6399936; doi:10.1186/s40035-019-0149-9)
Supplement: Supplementary file 1 — Table S1. Antibodies employed in this study. (DOCX 206 kb) [file 40035_2019_149_MOESM1_ESM.docx]

**Table. S1**

**
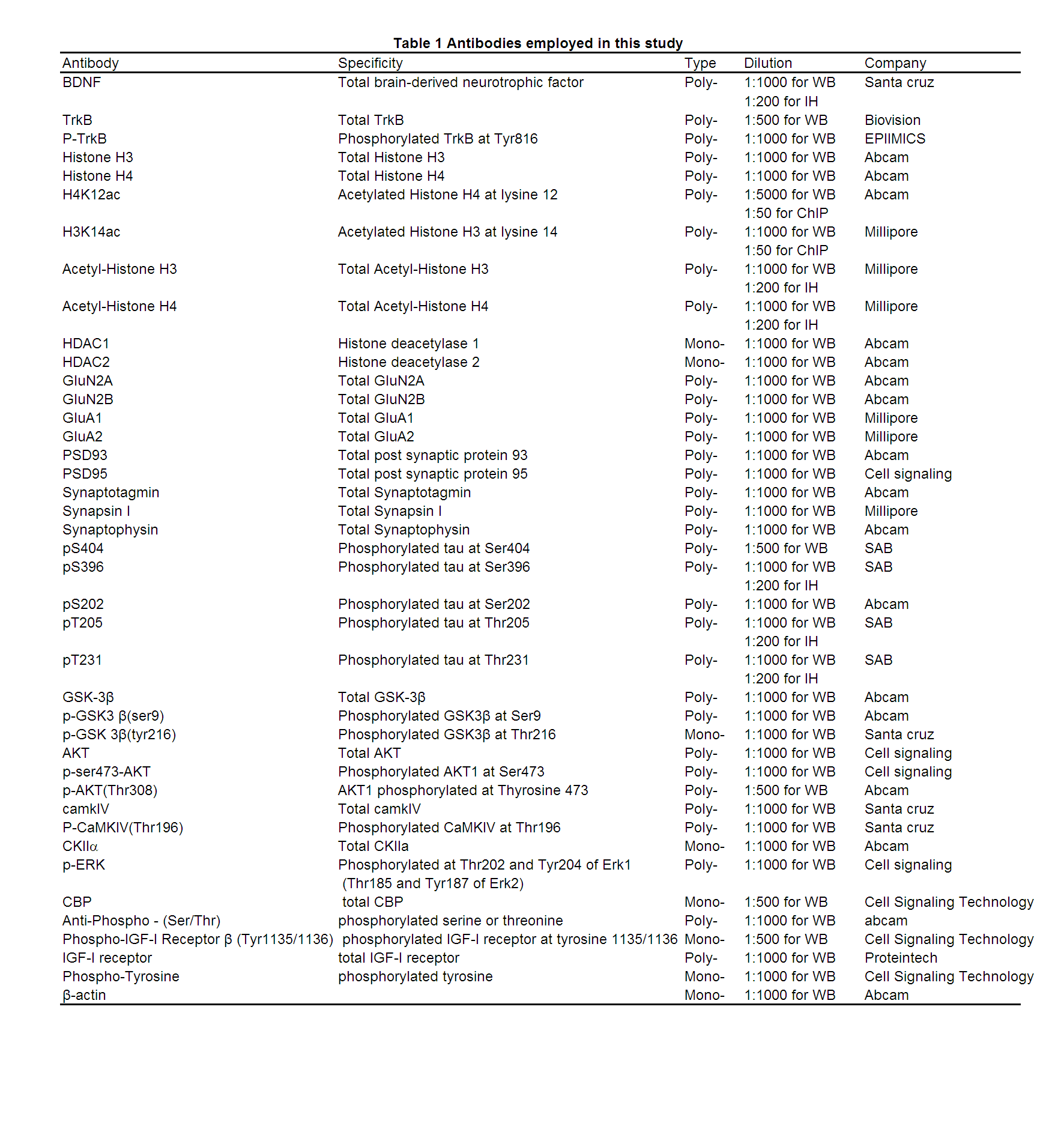
**

Mono: monoclonal, poly: polyclonal, WB: Western blotting, IH: immunohistochemistry, ChIP: Chromatin immunoprecipitation, IP: immunoprecipitation
