# Supplementary material for: Enriched gestation activates the IGF pathway to evoke embryo-adult benefits to prevent Alzheimer’s disease
Source: Transl Neurodegener. 2019 Mar 5;8:8. doi: 10.1186/s40035-019-0149-9 (PMC6399936; doi:10.1186/s40035-019-0149-9)
Supplement: Supplementary file 2 — Figure S1. GEE improves spatial memory in offspring, as measured by contextual fear-conditioning. GEE was conducted as shown in Fig. 1a. (a) F1 offspring were raised in a standard environment from weaning until 7 m of age when memory was tested using contextual fear conditioning (FC). (b) GEE improved memory without changing learning ability in the offspring. Ctrl, n = 16, GEE, n = 11, unpaired t test with Welch’s correction. **P<0.01 versus Ctrl. Data are presented as the mean ± s.e.m. (DOCX 113 kb) [file 40035_2019_149_MOESM2_ESM.docx]

**Fig. S1**


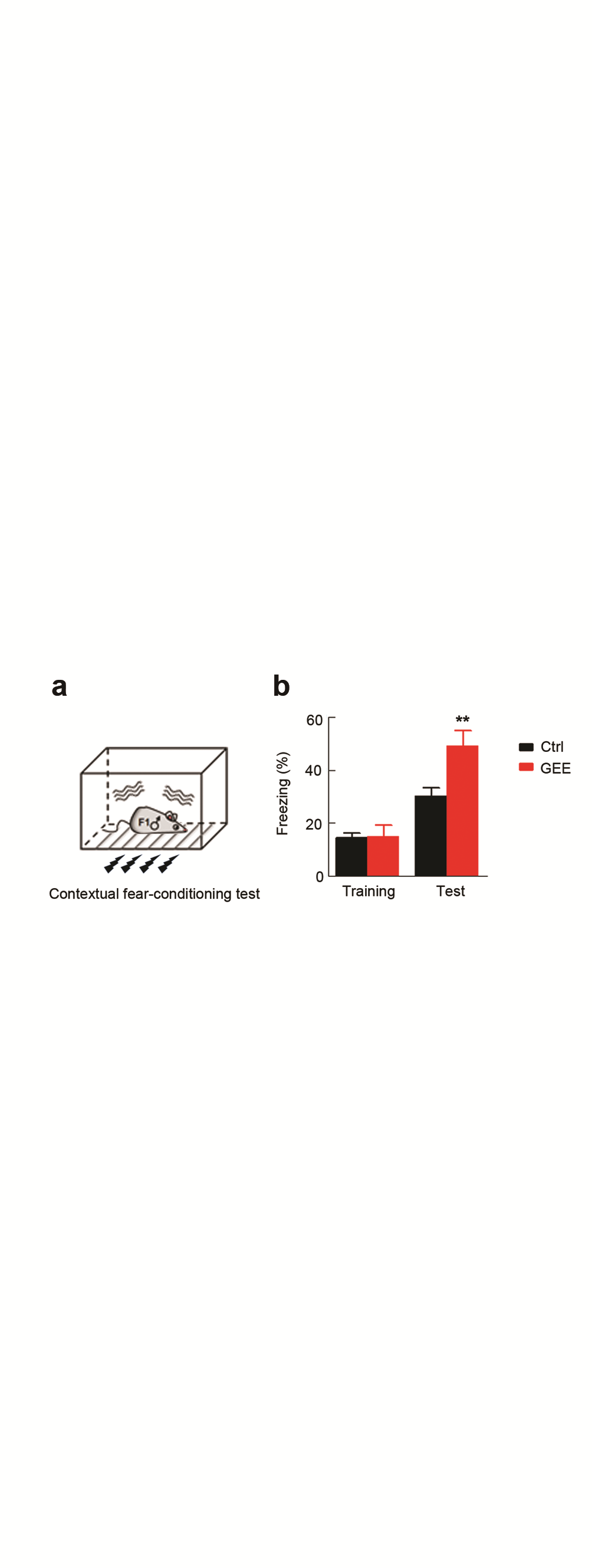


**Fig. S1. GEE improves spatial memory in offsprings measured by contextual fear-conditioning**. GEE was conducted as shown in Figure 1A. **(a)** F1 offerings were raised in a standard environment from weaning until 7 m-old when the memory was tested using contextual fear conditioning (FC). **(b)** GEE improved memory without changing learning ability in the offsprings. Ctrl, n=16, GEE, n=11, unpaired t test with Welch's correction. ***P*＜0.01 *versus* Ctrl. Data were presented as mean ± s.e.m.
