# Supplementary material for: Enriched gestation activates the IGF pathway to evoke embryo-adult benefits to prevent Alzheimer’s disease
Source: Transl Neurodegener. 2019 Mar 5;8:8. doi: 10.1186/s40035-019-0149-9 (PMC6399936; doi:10.1186/s40035-019-0149-9)
Supplement: Supplementary file 3 — Figure S2. GEE increases H3 and H4 acetylation in the offspring hippocampus. Representative immunofluorescence images stained using acetylated H3 (H3ac) and H4 (H4ac) in 7-m-old offspring hippocampal subsets (CA1, CA3 and DG). Scale bars, 50 μm. (DOCX 522 kb) [file 40035_2019_149_MOESM3_ESM.docx]

**Fig. S2**


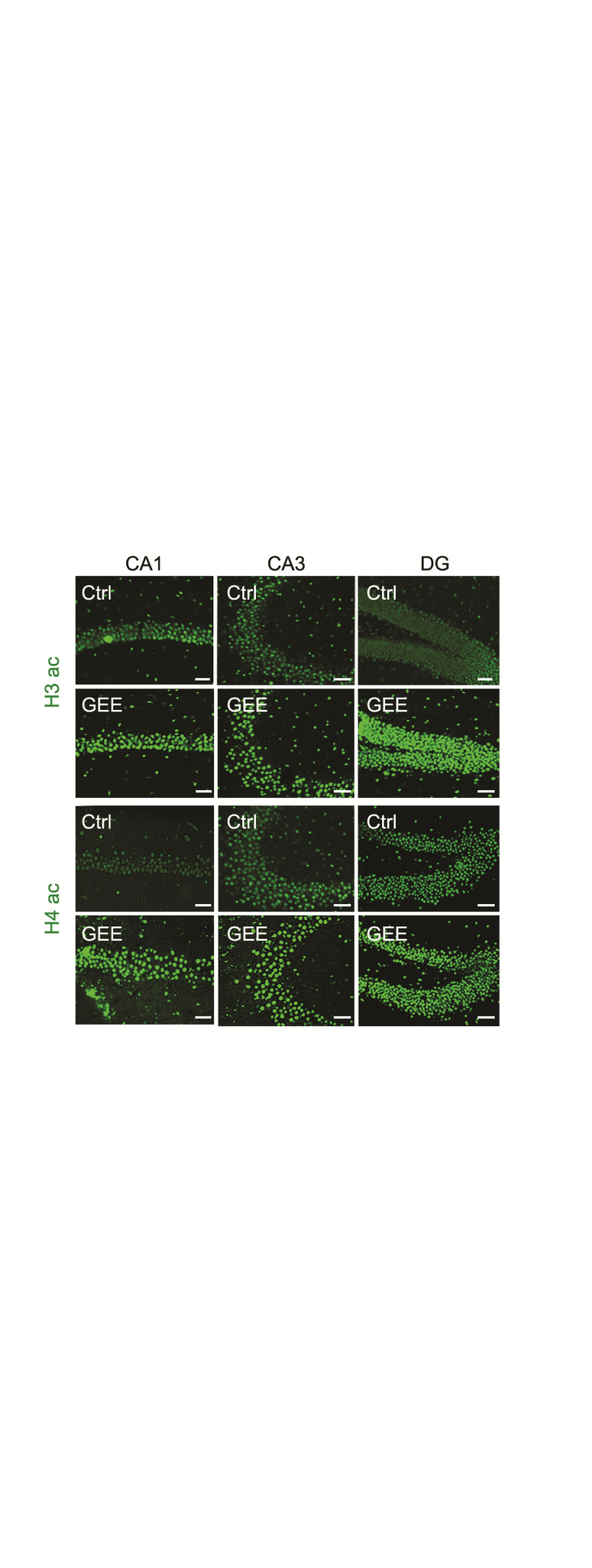


**Fig. S2: GEE increases H3 and H4 acetylation in offsprings’ hippocampus.** The representative immunofluorescence images stained by using acetylated H3 (H3ac) and H4 (H4ac) in 7 m-old offsprings’ hippocampal subsets (CA1, CA3 and DG). Scale bars, 50 μm.
