# Supplementary material for: Enriched gestation activates the IGF pathway to evoke embryo-adult benefits to prevent Alzheimer’s disease
Source: Transl Neurodegener. 2019 Mar 5;8:8. doi: 10.1186/s40035-019-0149-9 (PMC6399936; doi:10.1186/s40035-019-0149-9)
Supplement: Supplementary file 4 — Figure S3. Inhibition of HAT during GEE exposure has no effect on embryo weight. Embryos were weighed at E19 after GEE exposure with or without C646 treatment (HAT inhibitor). No differences in embryo weights were detected among the groups. n = 8–15 per group, one–way ANOVA, Tukey’s multiple comparisons test. Data are presented as the mean ± s.e.m. (DOCX 32 kb) [file 40035_2019_149_MOESM4_ESM.docx]

**Fig. S3**

**
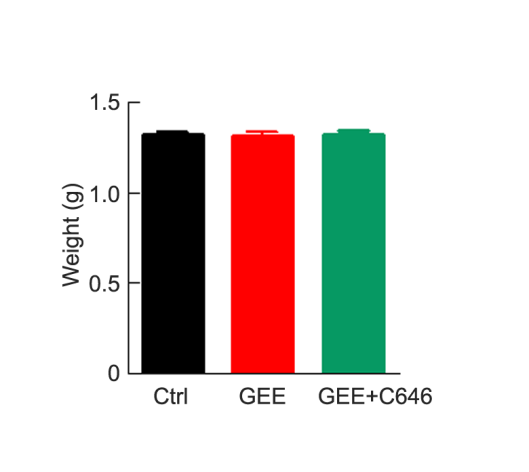
**

**Fig. S3: Inhibiting HAT during GEE exposure has no effect on embryos’ weight.** Embryos were weighed at E19 after GEE exposure with or without C646 treatment (HAT inhibitor). No difference of the embryo weights were detected among the groups. n=8-15 per group, One–way ANOVA, Tukey's multiple comparisons test. Data were presented as mean ± s.e.m.
