# Supplementary material for: Enriched gestation activates the IGF pathway to evoke embryo-adult benefits to prevent Alzheimer’s disease
Source: Transl Neurodegener. 2019 Mar 5;8:8. doi: 10.1186/s40035-019-0149-9 (PMC6399936; doi:10.1186/s40035-019-0149-9)
Supplement: Supplementary file 5 — Figure S4. Hippocampal HAT but not HDAC activity is positively correlated with spatial memory performance after GEE treatment. (a-c) Pearson analyses show the positive correlation of HAT activity with time spent in the target quadrant and platform crossings in the MWM test, and the freezing time in FC (for original data, see Fig. 1 and Figure S1). (d-e) No correlation was detected between hippocampal HDAC activity and partial memory performance after GEE treatment. Data are presented as the mean ± s.e.m. (DOCX 183 kb) [file 40035_2019_149_MOESM5_ESM.docx]

**Fig. S4**


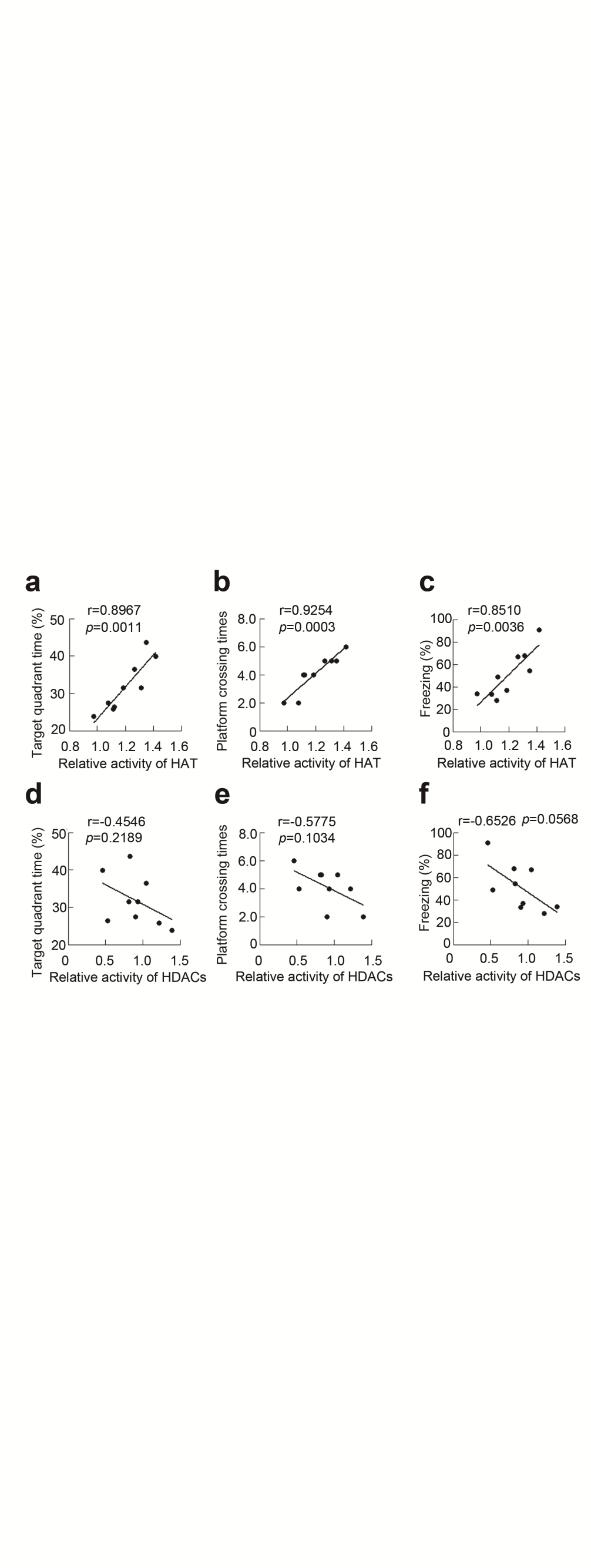


**Fig. S4. Hippocampal HAT not HDACs activity is positively correlated with spatial memory performance after GEE treatment.** (**a-c**) Pearson analyses show positive correlation of HAT activity with time spent in target quadrant and the platform crossings in MWM test, and the freezing time in FC(for original data, see Fig. 1 and Fig. S1). (**d-e**) No correlation was detected between hippocampal HDACs activity and patial memory performance after GEE treatment. Data were presented as mean ± s.e.m.
