# Supplementary material for: Enriched gestation activates the IGF pathway to evoke embryo-adult benefits to prevent Alzheimer’s disease
Source: Transl Neurodegener. 2019 Mar 5;8:8. doi: 10.1186/s40035-019-0149-9 (PMC6399936; doi:10.1186/s40035-019-0149-9)
Supplement: Supplementary file 6 — Figure S5. GEE decreases tau phosphorylation in offspring hippocampus. Representative immunohistochemical images of phosphorylated tau at Ser396 and Thr231 in hippocampal subsets (CA1, CA3 and DG). Scale bars, 50 μm. (DOCX 2457 kb) [file 40035_2019_149_MOESM6_ESM.docx]

**Fig. S5**


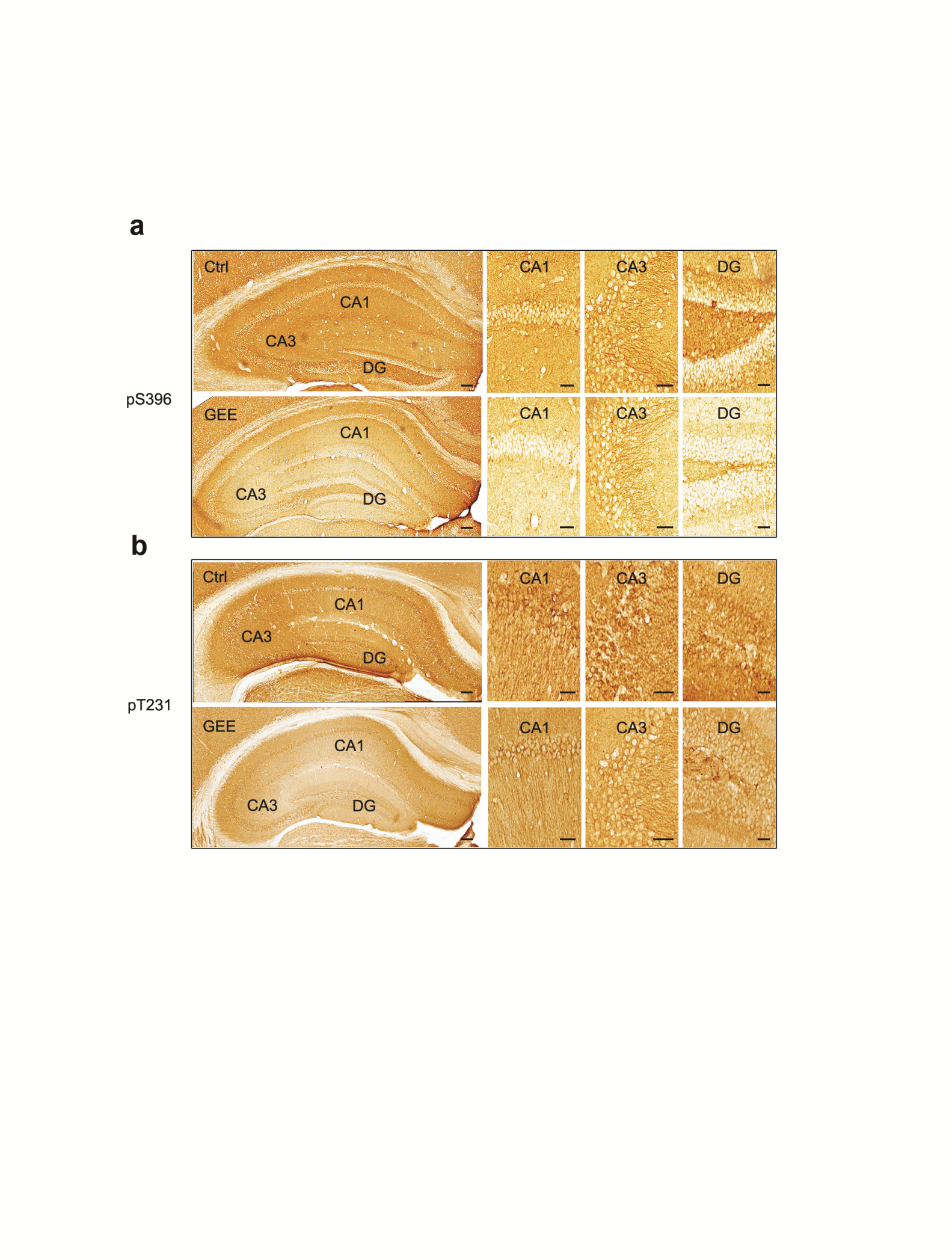


**Fig. S5: GEE decreases tau phosphorylation in offsprings’ hippocampus.** The representative immunohistochemical images of the phosphorylated tau at Ser396 and Thr231 in hippocampal subsets (CA1, CA3 and DG). Scale bars, 50 μm.
